# Wireless Body Sensor Communication Systems Based on UWB and IBC Technologies: State-of-the-Art and Open Challenges

**DOI:** 10.3390/s20123587

**Published:** 2020-06-25

**Authors:** Ivana Čuljak, Željka Lučev Vasić, Hrvoje Mihaldinec, Hrvoje Džapo

**Affiliations:** Faculty of Electrical Engineering and Computing, University of Zagreb, 10000 Zagreb, Croatia; ivana.culjak@fer.hr (I.Č.); hrvoje.mihaldinec@fer.hr (H.M.)

**Keywords:** wireless body area networks, wearable systems, ultra-wideband communication, intrabody communication, wireless body sensors, implanted sensors, vital signs monitoring, human motion tracking

## Abstract

In recent years there has been an increasing need for miniature, low-cost, commercially accessible, and user-friendly sensor solutions for wireless body area networks (WBAN), which has led to the adoption of new physical communication interfaces providing distinctive advantages over traditional wireless technologies. Ultra-wideband (UWB) and intrabody communication (IBC) have been the subject of intensive research in recent years due to their promising characteristics as means for short-range, low-power, and low-data-rate wireless interfaces for interconnection of various sensors and devices placed on, inside, or in the close vicinity of the human body. The need for safe and standardized solutions has resulted in the development of two relevant standards, IEEE 802.15.4 (for UWB) and IEEE 802.15.6 (for UWB and IBC), respectively. This paper presents an in-depth overview of recent studies and advances in the field of application of UWB and IBC technologies for wireless body sensor communication systems.

## 1. Introduction

**Figure 1:**
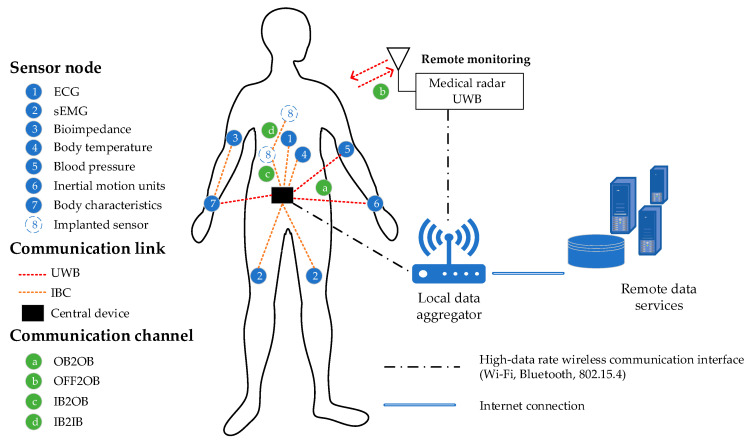
Wireless sensor network (WSN) topology of ultra-wideband (UWB) and intrabody communication (IBC) wireless body area network (WBAN).

Body area networks provide wireless connections between devices that operate within restricted space around a human body. They can be classified as *off-body* (communication among devices or systems which are in the vicinity of the human body and devices placed on the body), *on-body* (communications among devices and systems placed on the body) and *in-body* (communications to medical implants and sensors) networks [1,2]. An increasing need for accessible, miniature, low-cost, and user-friendly systems for wireless body area networks lead to new research topics, ultra-wideband (UWB) communication [3,4,5,6,7,8,9,10,11,12] and intrabody communication (IBC) [13,14,15,16]. UWB and IBC techniques are employed for implementation of wireless body area networks (WBANs), and they are used as short-range low-power wireless communication interfaces and low data-rate transmission, regarding IEEE 802.15.4 (UWB (PHY—physical layer)) and IEEE 802.15.6 (UWB (PHY) and IBC (PHY)) standards, respectively. As a part of the WBAN, they interconnect low-data-rate sensors. Among other radio protocols, sensors can communicate through UWB or IBC links, as shown in Figure 1. The data from sensors are gathered by a local data aggregator through the high data rate wireless communication interface and from there further relayed to data storage and management system backend, implemented typically as a cloud service. The wireless sensor body network can be divided into the sensor and communication part, where sensors can collect physiological, biomechanical, and other body characteristics data.

The Federal Communications Commission (FCC) became in February 2002 the first regulatory authority to define UWB technology utilization rules. According to [17], UWB communication systems are defined as systems in which (1) the center frequency is higher than 2.5 GHz, with the minimum bandwidth of at least 500 MHz, or (2) the current spectrum bandwidth must be higher than 20% of its center frequency, when center frequency is lower than 2.5 GHz. Compared to the FCC, the European regulatory authority separates two frequency ranges: from 3.1 GHz up to 4.8 GHz and from 6 GHz up to 10.3 GHz. Characteristics of UWB systems, such as high-data-rate, immunity to fading, reduced power spectral density, centimeter-level location estimation, low-cost, and fine time resolution (large bandwidth), make this communication technology suitable for applications related to the WBAN domain [4,5,8,9]. Furthermore, due to the above-mentioned characteristics, the UWB signal does not cause significant interference to other systems operating in the vicinity and does not represent a threat to the patients’ safety [18]. Moreover, UWB transmission, unlike narrowband and broadband technologies, is not based on modulated sinusoidal carriers; therefore, in the architecture of transmitters and receivers, there is no need for components such as local oscillators, mixers for frequency transposition to the desired band in the radio frequency (RF) spectrum, and reconstruction on the receiver side. Also, due to the transmission of Gaussian low-power pulses, a power amplifier is not required in the UWB transmitter. Due to this significantly simpler hardware solution, UWB receivers and transmitters have smaller dimensions than typical transceivers and are significantly cheaper to produce. Such characteristics make them available in the market for commercial applications at low price. Lately, there has been a growing interest in research of applications of UWB systems in the health sector, e.g., for vital signs monitoring [11,19,20,21,22,23,24,25], breast tumor detection [10,26,27,28], and for the wireless capsule endoscopy [9,18,29,30,31]. Besides the communication interface, UWB can be used as a radar in remote sensing and imaging techniques [19,32,33,34].

In an IBC or body channel communication (BCC) system the human body behaves as a volume conductor and is used as a part of the communication channel between the transmitters and receivers placed on the surface of the skin, in its vicinity, or implanted inside the user’s body [13,35,36,37,38]. IBC systems use lower frequency band than standard wireless systems, and they have lower power consumption and lower range. IBC signals are not prone to interference from external RF devices and are mostly confined within the human body, thus also reducing the risk of unwanted signal interception and providing higher security [13,39]. Although the human body communication (HBC) physical layer in the IEEE 802.15.6 standard for wireless body area networks is defined for 21 MHz ± 5.25 MHz frequency band [40], IBC system characteristics are usually studied between 100 kHz and 100–160 MHz. The selection of an appropriate carrier frequency in IBC systems arises from a trade-off among several factors: application in use, type of signal coupling, safety regulations to avoid interference with common biological signals, requirements for very low power consumption and high tissue conductivity, external noise, and so forth. As opposed to standard wireless systems, which require antennas for communications, IBC systems require only small electrodes. Signal and ground electrodes can be connected to the body, but they can also be left floating, depending on the signal frequency, coupling technique, and application [14,41,42].

However, aforementioned systems have their limitations, such as in modeling the propagation channel, random body movements, or UWB antenna design. Therefore, this paper provides an overview of both technologies, UWB and IBC, and recent advances in research. The content of this paper is presented as follows: in Section 2, a brief overview of wireless body sensor (WBS) technologies and current IEEE standards and regulations for UWB and IBC is provided. Section 3 provides a detailed description of emerging technologies, i.e., UWB and IBC. Finally, Section 4 brings the discussion and the conclusion.

## 2. State of the Art: WBAN Communication Systems

In this section, an overview of the ongoing research in the WBAN domain is presented. Wireless communication as such is increasingly present in daily life and provides fast and secure data exchange. Wireless communication in the healthcare sector, among other benefits, increases the awareness of personal health status because it provides sufficient information through continuous health monitoring from home and does not require frequent visits to healthcare institutions [43].

Three sensor types are most commonly used in wireless body sensors networks. The first type is on-body sensors (OB), such as electrocardiograph (ECG), electromyography (EMG), bioimpedance device etc., as shown in Figure 1. They are placed in a direct contact with the human skin surface. The second type is in-body (IB) sensors, implanted inside a human body, such as wireless capsule endoscopy, pacemakers, etc. [30,44]. Finally, the third type is off-body (OFF) sensors, placed in the vicinity of the human body. Various vital parameters, body characteristics, and biomechanical activities from a human body can be measured by on-body sensors, such as ECG, EMG, respiration, temperature, blood pressure, inertial motion units (IMU) sensors etc. Architecture of the body sensors communication system consists of various radio protocols, such as Bluetooth (BT), Zigbee, HBC, UWB or wireless LAN (WLAN), as a short-range communication interface. The communication between local base stations (BS) for data collection and aggregation, and remote data services go through the Internet connection infrastructure. In this paper the focus will be on the emerging short-range communication technologies, more precisely, ultra-wideband (UWB) and intra-body communications (IBC).

In general, in WBANs the vital and biomechanical parameters are collected by means of the various body sensors placed on or inside the body. Among conventional sensors for vital parameters, body characteristics, or human motion tracking (HMT), there is a growing need for an optimized solution that unifies sensing and communication functions in a single package. In-body to on-body sensors communication is very challenging because the human body represents a lossy communication medium which is highly frequency-dependent. Therefore, for safe and reliable communication interface from in-body to on-body it is necessary to provide an effective and suitable wireless solution [45]. UWB technology is a possible candidate due to its particular characteristics [3]. Low power spectral density (PSD) of the UWB transmission signal can be used in healthcare applications because low levels ensure the safety of human tissues, enabling high data rates and relatively long communication range at the same time. Several commercially available UWB monitoring devices appeared in the literature [23,46,47,48,49,50].

In WBAN applications, the IEEE 802.15.4 or IEEE 802.15.6 communication standards are the ones mostly used [2,51,52]. In [2] an overview of the on-body communication technologies, existing implementation and key parameters comparison of standards for wearable wireless networks are given. The IEEE 802.15.4 standard [53] provides a low-power, low-data-rate, and short-range communication. In 2007, UWB communication has been standardized as a part of IEEE 802.15.4 (upgrade version IEEE 802.15.4a [54]) standard and it provides the possibility to use an alternative UWB physical layer. A wireless body sensor network usually consists of various on- and in-body sensors, which communicate with each other by some short-range communication protocol. Due to the growing need for daily long term use and wireless monitoring of human body parameters, the IEEE 802 established in 2012 the new standard for the WBAN applications, IEEE 802.15.6 [40]. In the following subsections, more details about the aforementioned IEEE standards will be provided, in regard to the UWB and IBC physical layer.

### 2.1. IEEE 802.15.4a Standard (UWB (PHY))

The FCC was the first worldwide regulatory body which allowed legal use of the UWB technology in United States in 2002. The problem of spectral bandwidth and interference with other narrowband communications has been approached by limiting the maximum PSD in the entire frequency range of the UWB signal below a level that will adversely affect other communication protocols whose bands overlap with the UWB frequency range, e.g., Wi-Fi, Bluetooth, etc. Unlike narrowband communications, where a drastic reduction in power in a narrow spectral range would make communication significantly more difficult because almost all communication energy is contained within the narrow range around the central carrier frequency, such limitations for UWB technology are acceptable because the energy is evenly distributed across the wide spectral range. The FCC defined that the power spectral density must not exceed the prescribed maximum reference value of −41.3 dBm/MHz for the frequency range 3.1–10.6 GHz, and must be lower outside this range, depending on the specific application. Rules also define the operating range of UWB communication within the 7.5 GHz bandwidth. The stated PSD limit of −41.3 dBm/MHz refers to the measure of equivalent isotropically radiated power (EIRP). The EIRP is a measure of the radiated power from an ideal isotropic antenna in one direction, where the isotropic antenna is an antenna that radiates the same amount of energy in all directions. Real antennas do not radiate equally in all directions, so, to define the EIRP, the radiated power in a direction of maximum antenna gain is observed. The stated limit for PSD is relatively low and represents the limit that devices that are not intended for communication, such as computer peripheral monitors, have on the radiated interference in the radio frequency range. FCC regulations do not limit the radiated power by setting a simple flat limit over the entire spectral range for UWB communication, but for different sub-bands allowed limits of radiated power are defined by a spectral mask, which differs for devices used in indoors and outdoors areas. FCC regulations define different spectral masks for different classes of UWB devices, such as medical devices, surveillance devices, vehicle radar systems, ground-penetrating radars (GPR), etc. In addition, in 2007, the European Communications Committee (ECC) enacted regulations similar to those in the United States, but with somewhat stricter requirements.

First steps towards the standardization of UWB technology were made by the Institute of Electrical and Electronics Engineers (IEEE), by undertaking an effort to include UWB as a new physical (PHY) layer within the IEEE 802.15 standard. IEEE 802.15 is a working group dealing with standards in the field of wireless personal area networks (WPAN), with two subgroups being the most important for UWB technology, IEEE 802.15.3 (MAC (Medium Access Control) and PHY layers standards for high-speed WPAN networks, 11–15 Mb/s) and IEEE 802.15.4 (MAC and PHY standards for slow WPAN networks, which support various protocols often used nowadays, such as ZigBee, Thread, 6LoWPAN, WirelessHART, etc.). The standardization of the UWB PHY layer first moved in a direction of expanding IEEE 802.15.3a for high-speed WPAN networks, narrowing the choice to two proposed variants of UWB communication, multiband orthogonal frequency division multiplexing (MBOFDM) and direct-sequence UWB (DS-UWB). However, the working group failed to reach an agreement that would benefit from above proposals, and, finally in 2006, work on the standardization of UWB communications under the IEEE 802.15.3 standard was formally discontinued.

UWB communication has been standardized as a part of an upgrade of the original IEEE 802.15.4 standard to the IEEE 802.15.4a version, which provides for the possibility of additional use of an alternative UWB physical layer [55]. This version of the standard provides, in addition to low-speed data communication, the possibility of positioning with very high accuracy. IEEE 802.15.4a is also the first UWB standard that provides the possibility of wireless localization and supports one-way and two-way communication protocols. The IEEE 802.15.4a proposal was officially approved in 2007 by the IEEE Standards Association (the standard is called by its full formal name IEEE 802.15.4a-2007). The IEEE 802.15.4a standard specifies two options for generating communication signals: IR-UWB (impulse radio) and CSS (chirp spread spectrum). Both signal generation methods can be used for communication and IR-UWB also for precise positioning. The standard also defines three frequency bands:

- Sub–GHz: 250–750 MHz
- Low-band: 3.244–4.742 GHz
- Hi-band: 5.944–10.234 GHz

For all three bands, the standard defines 16 available channels, one in the sub-GHz band, four in the low-band, and eleven in the high-band. The frequency range of the channels is from 499.2 MHz to 1357.97 MHz, and the standard also defines which channels UWB devices operating in any of the listed bands must implement in communication, and which are optional. The physical UWB layer of the IEEE 802.15.4a standard supports various predefined communication data rates (110 kbps, 850 kbps, 1.7 Mbps, 6.81 Mbps and 27.24 Mbps). Standardized values of pulse repetition frequency (PRF) are also defined when sending pulses in a packet. Standardized PRF values of 3.9 MHz, 15.6 MHz and 62.4 MHz were defined. Additionally, within the IEEE 802.15.4a standard, standardized communication channel models are defined and used in the analysis of UWB signal propagation [54].

### 2.2. IEEE 802.15.6 Standard

In 2012, an international standard IEEE 802.15.6 for WBAN has been published, [40,56]. It applies to a short range (i.e., about human body range), low-power and highly reliable wireless communication for use in a close proximity to or inside a human body. It covers wide application areas, but the initial target applications were in wearable healthcare and mobile entertainment. The standard sets stringent requirements associated with WBAN transceivers, such as energy efficiency, interference rejection, low cost, quality-of-service (QoS) scalability, network coexistence, and safety. It also provides safe power levels for human body exposure. In each WBAN there is a single main hub and between 1 and 64 nodes. IEEE 802.15.6 standard defines one common media access control (MAC) layer for three physical layers (PHY), namely: narrowband (NB), UWB, and human body communication (HBC). Narrowband PHY is intended to be used by highly reliable short range wireless medical applications. Starting from 402 MHz to 2483.5 MHz, seven frequency bands, with channel bandwidths from 300 kHz to 1 MHz, information data rate between 75.9 kbps and 971.4 kbps, and receiver sensitivity between −95 dBm and −82 dBm are proposed, [40,56]. The first frequency band, between 402 MHz and 405 MHz, is dedicated to a communication with implanted devices (where at least one end of the wireless link is in the human body), while the remaining frequency bands are to be used by wearable devices. The wideband physical layer UWB PHY is based on the UWB technology, in particular IR-UWB and wideband FM (FM-UWB). It employs 11 operating frequency channels, each with 499.2 MHz bandwidth, and central frequency from 3494.4 MHz to 9984.0 MHz, grouped in low (3494.4 MHz, 3993.6 MHz, 4492.8 MHz) and high (6489.6 MHz, 6988.8 MHz, 7488.0 MHz, 7987.2 MHz, 8486.4 MHz, 8985.6 MHz, 9484.8 MHz, 9984.0 MHz) band groups. Channels 1 (3993.6 MHz) and 6 (7987.2 MHz) are mandatory for devices that implement the low band and the high band, respectively, while the remaining channels are optional. Mandatory data rate is 487.5 kbps for IR-UWB and 250 kbps for FM-UWB, but it can go up to 15.6 Mbps. Two modes of operation are the default mode, used in medical and non-medical applications and high QoS mode, used for high priority medical applications. HBC uses 21 MHz frequency band with 5.25 MHz bandwidth, and required data rates are 164 kbps, 328 kbps, 656 kbps, and 1.3125 Mbps with high required receiver sensitivity (−97.35 dBm, −94.34 dBm, −91.33 dBm, −88.32 dBm, respectively). The proposal was designed for exchanging data between devices by means of touching, through the body of a user.

Comparing IEEE 802.15.4 and 802.15.6 standards, with regard to UWB and IBC, one can see that they differ mainly by used frequencies, achievable data rates, and covered range [40,51,53]. IEEE 802.15.6 standard is designed to be used at shorter distances (human body size), meaning it has inherently lower power requirements and lower interference and is therefore safer to be used near a human body than the IEEE 802.15.4 standard. Unlike IEEE 802.15.4 (9 octets length), the transmitted data using IEEE 802.15.6 need to contain a MAC header with 7 octets length [51]. Currently, aforementioned standards are the only ones including impulse radio UWB PHY specifications, besides other PHYs [52]. In addition, by comparing IEEE 802.15.4 and IEEE 802.15.6-2012, the first evident difference is the absence of the sub-gigahertz band.

## 3. State of the Art: Emerging Technologies for the WBS Applications

### 3.1. State of the Art: Ultra-Wideband Communication Technology

UWB communication is based on sending very short pulses (of duration from 100 ps up to 1 ns) in time domain which occupy large bandwidth in the frequency domain. Due to its characteristics, UWB technology represents a viable solution for various applications, such as healthcare and precise localization in indoor environments. It can be shown that the UWB technology is capable of high accuracy localization in closed spaces, and it is able to detect the micromovement of the displacement of the internal organs inside the human body [5,8,10,12,48,57,58]. Additional benefits are high communication data rate, low price, immunity to the multipath problem, and capability of localization and communication at the same time. UWB technology is based on an ultra-short signal that can penetrate obstructions (such as walls or human tissues) because its wide spectral usage minimizes the negative impact of frequency-dependent loss in communication media.

Unlike narrowband signals, UWB contains a wide range of frequencies, making it a potential candidate for various wireless communication systems applications. In UWB signals, the absolute bandwidth B is defined as the difference between the upper *f_H_* and the lower *f_L_* frequency in the signal spectrum at which the radiated signal power is 10 dB less than the power radiated at the maximum center frequency:

*B* = *f_H_* − *f_L_* (1)

UWB signals are declared as signals with ultra-short duration and localized at short time intervals in the time domain, typically in the nanosecond range. Contrary to narrowband signals, the UWB signal uses the entire spectrum width and does not have a precisely defined center frequency of the carrier signal. However, in a process of UWB signal generation it is possible to define a limited frequency range within which the signal will be contained. The aforementioned frequency band must meet the requirements of relevant standards, whereby the maximum of the PSD must be located in the center of the frequency range [59].

#### 3.1.1. UWB Communication

**Figure 2:**
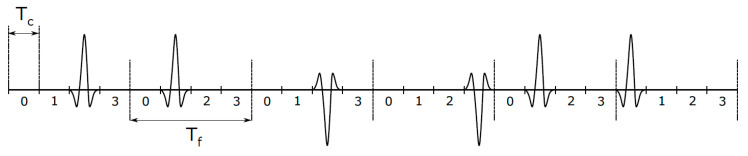
Impulse radio ultra-wideband (IR-UWB) signals in the time domain.

The UWB system typically transmits ultrashort pulses in a time domain with a small duty cycle. Therefore, it can be described as an IR-UWB system. Unlike narrowband radio communication systems, the IR-UWB transmits information by encoding via the position and polarity of pulses in the packet, as described in Figure 2. Figure 2 shows an example of the IR-UWB package (three bits’ information length) in the time domain. Each UWB pulse is located within a signal time frame, with the duration of the *T_f_*. In addition, each time frame is divided into smaller intervals, so-called chips, with duration *T_c_*. UWB pulse coding is performed by using a time-hopping (TH) scheme to reduce the pulse collision in situation where there are multiple UWB transmitters in the same area. As shown in Figure 2, the TH at the first bit is represented by the combination {2; 1}, because, in the first frame, the first pulse is shifted from the beginning of the frame by 2*T_c_*, and in the second frame the pulse is shifted by *T_c_*. If we take into account that, in the first and third frame, positive pulses are transmitted, and, in the second, negative pulses, the total information that is sent in this way via the IR-UWB communication channel is {+1; −1; +1}.

It is important to emphasize that the shape and width of the UWB pulse over time affects both the bandwidth and power spectrum density. Therefore, the design and generation of UWB pulses are one of the biggest challenges in the practical implementation of UWB technology. The second derivation of the Gaussian function, Hermite polynomials, or wavelets are most often used to generate UWB pulses. The second derivation of the Gaussian function is the most common choice because of its favorable properties [59].

Compared to the narrowband wireless communications, UWB has a lot of advantages due to the specificity of the signal it sends (localization of pulses in time) and wide frequency range:

- better signal penetration through various obstacles (e.g., walls in buildings, human tissues);
- the possibility of achieving high accuracy and precision of radiofrequency positioning due to communication using very narrow pulses in the time domain;
- high-speed data communication;
- low price and consumption.

In addition to the already mentioned IR-UWB approach, there are other possibilities for generating UWB signals. One approach is MB-UWB, where data are multiplexed into subcarriers along with the entire band from 3.1 GHz to 10.6 GHz, or in any other part thereof. In each sub-band, data are transmitted using orthogonal frequency division multiplexing (OFDM), called OFDM UWB communication mode. Also, in addition to UWB systems that operate with a low duty cycle, there are versions of UWB systems with the continuous transmission, e.g., direct-sequence code division multiple access (DS-CDMA).

The disadvantage of the IR-UWB system is the presence of a large number of multipath signals due to the reflection and scattering of UWB signal, i.e., the receiver receives a larger number of multipath components (MPC) instead of one isolated pulse. Effects that occur due to multilayer propagation cause major problems in the design of hardware that must be able to correctly detect and decode the received message. However, since ultrashort pulses are used in the time domain, it has been shown that in practice it is possible to implement a hardware that can perform efficient filtering of MPC components in the time domain and thus accurately detect the message [48,58,60,61]. If time between sending two pulses is greater than width of the impulse itself, the crosstalk effects between the symbol and the multipath signals are reduced or even eliminated. Therefore, this paper will primarily focus on IR-UWB systems due to growing usage of the IR-UWB technology in various research domains in the field of communication and localization, such as industrial, healthcare, and sports applications.

#### 3.1.2. UWB Signal Propagation Modeling

To understand the possibilities and ways how to use the UWB technology, it is necessary to understand the models that describe the propagation of UWB signals, as well as features of the communication channel. The modeling of communication channel characteristics differs significantly from narrowband technologies due to the fact that the signal contains a large range of spectral components in the frequency domain and an ultrashort duration of the impulses in the time domain.

**Figure 3:**
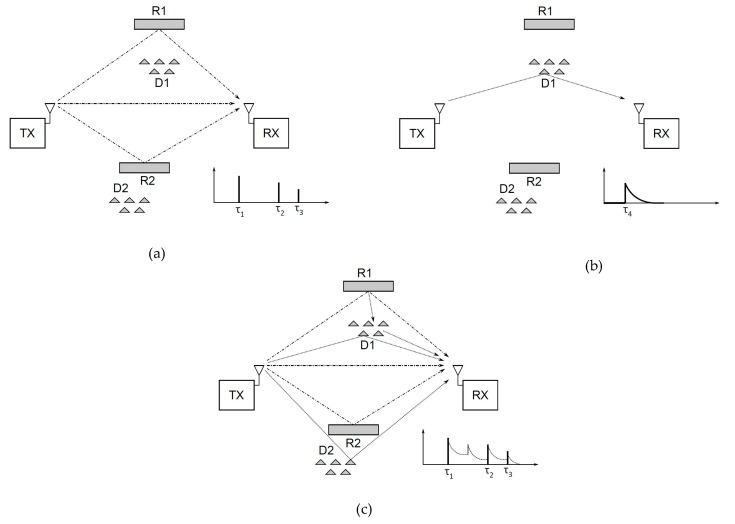
Multiple signal propagation in conditions of multiple reflections and scattering: (**a**) direct and specular reflective multipath components (MPC) components, (**b**) single MPC component generated by scattering, (**c**) overall MPC signal components on the receiver (RX); Tx—transmitter, R1, R2—reflectors, D1, D2—diffusors, τ—time.

The main parameters for modeling the UWB communication channel are path loss (*PL*) and power delay profile (PDP). In the UWB systems, the *PL* is described by the following equation:

*PL*(*d*) = *PL*_0_ − 10*n*log10(*d*/*d*_0_) (2)

where *d_0_* is the reference distance where the reference value *PL_0_* of the *PL* is calculated. On the other side, the PDP parameter provides information about time domain behavior of the received signal power *P(t)*, observed from the moment when the first MPC component of the signal reaches the receiver. For better understanding of the PDP meaning, it is necessary to understand causes of the multipath propagation of UWB signals and effects induced on the receiver side. Ideally, in a homogeneous media, the electromagnetic wave propagates directly from transmitter to the receiver, which receives only one signal component. In the case of IR-UWB communication, this would mean that the receiver receives only one UWB pulse sent from the transmitter. In lossy materials, such as human tissues or building walls, signal propagation is affected by various effects, such as reflection, diffraction, scattering, shadowing, etc. These effects cause the emergence of new components, which reach the receiver at different time instants. Figure 3 shows an example of multipath propagation in the IR-UWB communication, where in addition to effects of the reflection, effects of the signal scattering are also taken into account. In specular reflection, the beam is reflected from the reflector, but the reflected beam is not divided into more beams. In scattering, the incident beam is divided into a number of reflected beams, all of which do not have to be reflected at the same angle. In a process of reflection, additional changes in the amplitude, phase and delay of the signal may be introduced. Effects of scattering on a particular material are frequency dependent, so, in case of a UWB signal, the beam will split into a larger number of scattered components.

To observe multipath effects of signal propagation in the modeling of wireless communication channels, the channel impulse response (CIR) is used, described as:

(3) $$h\left(t\right)=\overset{\infty }{\underset{k=0}{\sum }}h_{k}\delta \left(t-\tau_{k}\right)$$

where $h_{k}$ and $\tau_{k}$ are amplitude and delay of the *k*-th component in multipath fading. The model described in (3) is applicable for cases where multipath components occur due to, for example, specular reflections, in which MPC components represent a delayed and amplitude modulated replica of the original pulse. Model (3) is typical for narrowband signals because properties of materials are constant in a sufficiently narrow frequency band around the carrier frequency.

However, in UWB communication, due to the very wide spectral content of components in the signal, MPC components can be scattered in the time domain. As a result, MPC components in UWB signals come in clusters, which is why the Saleh–Valenzuela (SV) model is used to model characteristics of UWB channels, and CIR for the SV model is described as:

(4) $$h\left(t\right)=\overset{K}{\underset{k=0}{\sum }}\overset{L_{k}}{\underset{l=0}{\sum }}\alpha_{k,l}e^{j\theta_{k,l}}\delta (t-T_{k}-\tau_{k,l})$$

where *K* is number of clusters, *L_k_* number of MPC components in the cluster, $\theta_{k,l}\;$ is channel coefficient for *l*-th component of the *k*-th cluster, *T_k_* is *k*-th cluster delay, $\tau_{k,l}\;$ is *l*-th beam delay in relation to *k*-th cluster, $\theta_{k,l}\;$ and is uniformly distributed phase on the interval [0,2π]. The PDP parameter describes the power level of the received signal *P(t)*, observed from the moment of receipt of the first MPC component of the signal at time τ=0. Observing the dependence of the PDP parameter in time, the channel impulse response *h*(*t*) can be calculated as the local mean value *|h(t)|*^2^. In the SV model of the UWB channels, arrival times of clusters and individual beams within them are modeled by a Poisson random variable. More details are provided in literature [62,63].

**Figure 4:**
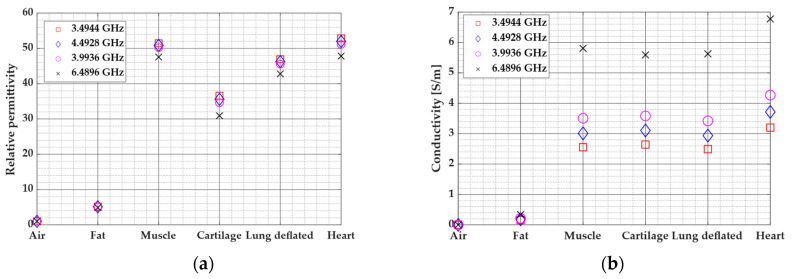
Relative permittivity Ɛ (**a**) and conductivity σ (**b**) through the five tissue layers for the four UWB communication channels with the 3.4944 GHz, 3.9936 GHz, 4.4928 GHz and 6.4896 GHz center frequency with the bandwidth 500 MHz.

Among aforementioned parameters, in the case of measurements on the human body, there are a few more parameters that have impact on the UWB radio channel. Besides demographic characteristics (sex, age), the human body position has impact on the radio channel, as well as measurement environment, antenna position and implants inside the human body [3,7,8,10,64,65,66,67]. In addition, it is necessary to classify the channel dependence on the position of systems during the experiment as a line-of-sight (LOS), non-line-of-sight (NLOS), or partial non-line-of-sight. Furthermore, it is also necessary to investigate the influence of the position of a wearable antenna placed on the human body [5,67,68]. In [5], the authors showed that the forehead location provides the best range estimate in multipath conditions. They used seven wearable antenna locations (forehead, hand, chest, wrist, arm, and ankle) in the measurement environment. The conclusion is that the type of a channel link and a position of wearable antennas lead to significant changes in the radio channel link. As explained in [69], different postures of the arm have influence on the channel impulse response (CIR) and define whether the channel between two nodes are of a LOS or NLOS type. Moreover, human tissues are lossy material and therefore different age and sex have the influence on tissues structures inside a body, more precisely, they have different relative dielectric properties [3,66]. Beside the real part of the permittivity, lossy materials may have an imaginary part that significantly influences the calculation of propagation velocity, attenuation, reflection and transmission parameters [70]. In research of applicability of UWB technology for determination of the heart rate (HR), the observed UWB signal propagated through five different tissue layers. The thickness of each tissue layer is different, along with the effect on characteristics of the observed propagation channel. The paper [70] presented the multilayer model of electromagnetic signal propagation through the human body. EM wave propagation simulation through human body tissues by means of Gabriel et al. [71] showed that, for higher frequencies, the conductivity σ increases linearly while relative permittivity Ɛ decreases linearly, as shown in Figure 4. Therefore, it is important to define and understand related channel propagation parameters before placing communication systems on the human body as a part of WBANs.

UWB radar measurements in most cases are carried out by placing the UWB radar in front of the subject in a sitting position. During the measurement, the subject is asked not to make sudden movements so that there are no additional disturbances caused by the subject movements. Also, it should be emphasized that the antenna must not be in a contact with the skin due to dielectric properties of human tissue and therefore there must be a sufficient gap between the antenna and the skin, which can be filled with a ceramic substrate with small losses.

In [72], Staderini depicted the difference in reflection magnitude between the heart muscle and the blood that circulated through it. It was concluded that approximately 10% reflection magnitude of the radio frequency energy could be expected at the heart’s muscle–blood boundary. Furthermore, it was shown that the main advantages of the UWB technology and its application in monitoring health condition are monitoring of the internal organ movements without direct skin contact, and ability to work through clothes at the distance of up to several meters [72]. The body surface creates a large reflecting surface between the air–skin boundary. The coefficient of reflection is approximately 70 up to 80%, and blood, muscles, and bone tissue result in higher signal attenuation, unlike the spinal cord and bones. Breathing causes chest movement in range from one up to a few millimeters and can be also monitored by UWB radar approach [7,66].

#### 3.1.3. UWB Applications and Systems

In this section, some typical UWB usage scenarios and systems in the context of WBAN applications will be presented. The first part describes some examples of UWB applications in the healthcare domain while the second part shows an overview of UWB technology in the human motion tracking (HMT) systems.

Within the last couple of years, there has been a large growth of the usage of UWB technology in healthcare applications, more precisely, in the area of vital sign detection and monitoring. The working principle of the existing radar-based approaches is based on the electromagnetic energy propagated towards and through the body and its reflection on border surfaces between tissues with different wave propagation properties [20,21,22,24,32,47,73,74,75,76,77,78,79,80,81,82,83,84]. Lately, another approach based on the transversal propagation method proved to be an alternative viable solution for using UWB in vital signs monitoring applications [85,86,87]. It is based on the assumption that heart and lung motions are continuously changing the communication channel properties and modulating the observed signal power on the receiver side [85]. During the measurement the transmitted signal power and relative position of transmitter and receiver units do not change over time. Furthermore, the UWB communications are also used for implantable sensors, for example, wireless capsule endoscopy [29,30,31,88,89].

Likewise, in applications on human motion tracking the UWB technology can be used in two ways. The first approach is to use the radar principle, where UWB channel is analyzed to detect, track or classify human activity in closed spaces or even behind walls [58,90,91,92,93,94,95,96,97]. Another possibility is to measure the propagation time of UWB signal from transmitters placed on tracked objects or parts of the human body to stationary BS receivers [48,49,50,57,98,99,100].

##### UWB Applications in Healthcare

The research on the applicability of UWB-based technologies in healthcare can be divided into several approaches, depending on the monitored parameters. The most frequent application of UWB technology is vital signs monitoring, i.e., respiration rate (RR) and heart rate (HR) [12,19,25,78,79,82,101]. The most recent research focus on wireless in-body to on-body UWB communication, i.e., capsule endoscopy and tumor detection [28,29,30,31,88,89]. However, there is an increasing tendency that UWB technology, in addition to the sensing part, could be also used as a communication interface, thus meaning that only one sensor node is necessary for providing information about the human health status and for communication with an external base station.

In most cases, vital signs monitoring is performed by using the UWB radar approach, as shown in Table 1. The radar approach is carried out by placing the radar at a fixed distance in front of the human body. The most common radar used in the studies is the IR-UWB radar. There are particular advantages of using UWB technology for implementation of the radar method for vital signs monitoring. It is possible to implement a miniature transceiver that can be used as a biomedical sensor for the heart and lung displacement detection. One of first studies in using the UWB radar for vital signs monitoring was presented by McEwan in [102,103]. In [72], a proposed model includes thickness, impedance, linear attenuation, and wave speed of six tissues taken into account for the heart movement detection. Furthermore, one application of the IR-UWB radar is vital signs monitoring through walls or similar obstacles, i.e., when there is no direct line of sight. Individuals can be detected through the wall without mobile stations using UWB technology as a detector of respiration, or chest displacement. By using a UWB radar in the 5 GHz band, it is possible to detect repetitive frequency components in the range 0.2–0.7 Hz that correspond to the human respiration rate. In addition, researchers proposed UWB systems for monitoring vital signs in cars while driving [104]. What all stated studies have in common is that they were performed in controlled conditions and there is no sufficient investigation of the random body movement during the monitoring process. These researches are usually based on the last recorded signal in the stationary conditions.

Recent research on UWB technology as a communication interface in WBAN systems, with an emphasis on in-body to on-body systems, is shown in Table 2. As presented in Table 2, there is an increasing number of studies of UWB applications in wireless capsule endoscopy [9,30,88,105,106] and implanted devices in general [3,107]. Due to its characteristics, UWB is suitable for implanted applications since it enables low power transmission and small physical dimension. Perez-Simbor et al. [88] presented one of the first researches of the path loss model for the UWB frequency in the gastrointestinal (GI) scenario. In addition, they performed studies in simulations, phantoms, and in vivo measurements. Leelatien et al. [3] investigated a possibility of wireless monitoring of transplanted organs (liver). They concluded that it is possible to monitor with attenuation variation of 30 dB, with respect to 40 mm of the largest organ movement distance due to respiration-induced organ movement. Särestöniemi et al. [7] research of the human abdomen area UWB on-body radio channel characteristics concluded that if the antenna separation distance is large, on-body channel characteristics vary significantly depending on the body size and shape of the subject. Furthermore, Särestöniemi et al. [30] provided studies on the UWB radio channel characteristics study between a capsule endoscope and a directive on-body antennas in different parts of the small intestine. For the study purpose, they used four anatomical voxel models from the electromagnetic simulation software CST Studio Suite. They investigated the difference obtained between two on-body antennas and bring power flow presentations of them. Also, they investigated the influence of the different rotation angles of the capsule and concluded that radio channel characteristics varied significantly depending on the capsule model and on-body antennas location. In addition, Song et al. [28] conducted research on breast tumor detectability. They showed that the IR-UWB radar-based detector is a potential candidate for early-stage breast cancer detection.

On the other side, one of examples of dynamic off-body communication UWB radio channels measurements for WBAN communication is given in [108]. Their focus was investigation and extraction of the human body shadowing effect, and thus measurements were done in an anechoic chamber. Measurements were carried out using two planar prototypes (dipole and double loop) antennas in the frequency range from 2–8 GHz and at six different on-body locations for the antenna’s placement, and one off-body antenna.

##### Application of the UWB in Human Motion Tracking

UWB technology for HMT can be used in two ways. The first approach is to analyze changes in the UWB channel to detect the presence [109,110], monitor motion [48,60,90,91,109,111] or even classify the human activity [112,113] in indoors environment or behind the obstacles, such as walls [92,114,115,116]. For instance, by monitoring the response in the UWB channel using the UWB radar method, it is even possible to detect balance, posture change, or oscillation while standing [57]. Localization is possible even behind walls with up to 55 cm of thickness, with an accuracy of 1.13 cm at a distance of 46 m [61]. By comparing reflections of UWB signals, it is even possible to recognize a person by their stature and gait [117]. Moreover, simulations showed that it is possible to determine the distance and direction of human movement with multiple static antennas with a maximum accuracy of 0.5 m [58]. One of the first investigations of off-body UWB channels for a body-centric system in an indoor environment and LOS/NLOS configurations was presented in paper [116]. They bring experiments for a body-centric fiber-optic-fed multichannel antenna transmitter and base station mounted on the wall. Also, they investigated mutual coupling effects on the received power diversity gain for on-body antennas. The advantages of this research that it collected empirical data, and so they capture real movements of the human body for body-centric systems. They concluded that received power is mainly affected by body shadowing.

While applications of the radar mode are diverse, the second approach for using UWB primarily refers to determining the time of arrival (TOA) of signals, i.e., positioning the UWB transceiver using a fixed infrastructure [49,94,95,98,99,118,119,120,121,122,123] or placing it on certain parts of the body for determination of relative distances between antennas [48,50,100,124,125]. In [68], it was shown that the commercially available module DWM1000 can be used while measuring rapid movement. The study was conducted with the aim of determining the optimal number of samples when averaging the measurements, taking into account different speeds of movement. A similar study of the application of UWB technology was conducted in [69], where an attempt was made to determine whether the solution based on DW1000 was suitable for use during sports activities. It is important to emphasize that the measurement system exhibits different behaviors in situations of higher and lower physical activity, which can be covered by appropriate kinematic models in order to improve the measurement results [99].

In cases when determination of the position in 3D space is important, improvements can be achieved by combining UWB positioning technology with inertial or other sensors [100,125]. Using sensor fusion methods, data collected from multiple sensors are jointly interpreted to take advantage of individual information sources [95,119,121].

In the basic design of the human gait monitoring system, [97] a UWB node with a DW1000 integrated circuit was placed on the subject’s foot. This simple system has been found to provide satisfactory directional accuracy for gait analysis in the spatial and temporal domains. The error in the direction of walking coordinates is between 19 mm and 23.14 mm. Unlike other more expensive systems, this lining does not require calibration or multiple numbers of nodes mounted on a human and makes it a good candidate for clinical gait trials. A similar system was proposed in [122], where it was expanded using IMU sensors.

The aforementioned DW1000 chip, manufactured by DecaWave, complies with the IEEE 802.15.4-2011 standard and enables the measurement of the time of sending and receiving UWB signals with a resolution of 15.6 ps, which means a precision of 10 cm in positioning applications [126]. At the same time, the module offers the possibility of high data rate (up to 6.8 Mb/s) which is achieved by a combination of BPM (burst position modulation) and BPSK (binary phase shift keying) modulation, with a declared range of 290 m in LOS conditions. High immunity to the problem of multipath signal propagation makes this platform an ideal choice for industrial and other environments where the RF signal is reflected on metal surfaces. According to the manufacturer’s specifications, it is possible to update the position up to 3.5 Hz for one moving tag.

It is worth mentioning that there are a number of other manufacturers that offer UWB solutions similar to Decawave’s, such as Ubisense [127,128], Humatics [129], or Blinksight [130], but on finished-product level for various industrial applications. Only BeSpoon [131] offers a similar development environment for researchers in the form of chip solution but, when compared with Decawave’s, its performance is slightly worse and significantly more unreliable [132]. This also can be noticed in the number of referenced researches that use DW1000 chip-based development kits (such as Pozyx [133]) as go-to platform for measurements.

High availability of ready-to-use UWB solutions caused an increase in the number of possible research fields. Generally, IMU sensors were used for measuring, detecting and classifying human motion or activity because of their low price, high availability and small size. Nevertheless, additional sources of information-rich data are required since the current approach caused a stall in human activity research [134]. Because of this, sensor fusion methods such as complementary and Kalman filters, with developed human motion models, became a primary research topic [118,121,128,135,136]. UWB is generally used as an additional correction factor for previously developed methods. However, in [137], the authors analyzed available commercial and academic solutions and concluded that they either focus on small scale applications or require infrastructure (either Ethernet or Wi-Fi) for scalability. Additional research, such as [137,138], is needed in protocol, wireless synchronization and infrastructure so that scalable and power efficient systems can be developed. Since UWB localization systems are based on timestamping exact moment of incoming and outgoing signals, extra research needs to focus on sources of error in ranging methods [139], as well as to compensation of clock drifts [140].

### 3.2. State of the Art: Intrabody Communication Technology

In this subsection, the overview of recent advances in IBC for wireless communication is presented. The two main methods of intrabody communication are galvanic and capacitive coupling. In a galvanic coupling method, both electrodes of IBC devices are in a direct contact with the human body. A single signal differential path is established through a current flow that penetrates into the tissue. Due to this characteristic, galvanic coupling has frequently been proposed as a viable alternative for the communication between implanted sensors [36,145]. In a capacitive coupling method, forward signal path is established through the human body and a return path is formed through the environment. This feature allows the interconnection of devices that are both deployed on the same body surface or close to it, without the need for direct contact with the skin, but can also be used for communication between implanted devices, as was proposed recently [146,147,148,149,150]. The capacitive method allows higher achievable data rates and lower path loss compared to the galvanic IBC method, especially for higher communication distances on the body.

In [151], authors analyzed compliance of the current density and electric/magnetic fields generated in different modalities of IBC with the established safety standards using the circuit and finite element method (FEM) based simulations. Results showed that currents and fields in the capacitive IBC system were orders of magnitude smaller than specified safety limits. However, galvanic HBC with differential excitation at the wrist for some cases resulted in localized current densities and field intensities around the electrode, which were significantly higher than safety limits. They also carried out a small in vivo study of vital parameters monitoring using capacitive IBC and the acquired data statistically showed no significant change in any of vital parameters of the subjects. Gao et al. [152] analyzed the safety of galvanic IBC using empirical FEM arm models based on the geometrical information of six subjects. They computed the electric field intensity and localized SAR and, in some cases, 2010 ICNIRP safety limits were exceeded. In order to comply with safety standards, for galvanic IBC they suggested using 100–300 kHz frequency signal, which allows the current signal of 1–10 mA and the voltage signal of 1–2 V.

#### 3.2.1. Measurements of IBC Channel Transmission Characteristics

Characteristics of IBC channels have already been studied extensively, but there are some challenges that still need to be addressed for optimal deployment of IBC technology. IBC channels change dynamically with electrode positions and size, subject, subject’s movements, and surrounding environment [13,15,41,153,154,155]. Establishing a proper procedure and measurement setup for measuring IBC channel characteristics, while keeping the overall IBC signal path intact, is a very challenging task [156,157], since introducing any kind of measuring equipment into the IBC channel modifies the return signal path and usually influences the measurement results. The use of different measurement equipment (signal generator, oscilloscope, network and spectrum analyzer), effects caused by different cables and connections, differences when using transformers or battery powered devices, the effect of the load resistance, and ground strategies were extensively studied in [13,153,158,159,160]. It was shown that using balun transformers and commercial equipment with 50 Ω input impedance resulted in higher measured gain than in a realistic IBC channel due to the improper ground isolation, and with lower gain at low frequencies due to the low frequency termination [159,160]. Also, devices with large physical size (like commercial network and spectrum analyzers) created a larger than expected return path, whether they were isolated with baluns or not, thereby increasing the measured channel gain [15,41,160,161,162,163]. In practical implementations, a galvanic decoupling between the human body and measuring instruments was realized using an optical link [164] or, more often, connecting balun transformers between the transmitter/receiver electrodes and the rest of the measuring equipment [15,16,153,155,158,160,162,163,165,166,167]. However, the influence of transformers on the measured results has been commented in a few papers only recently [15,40,119,124,126,129,131,132]. It was shown that the value of the capacitance between primary and secondary windings of the transformer could influence results drastically [164,166]. Furthermore, although inherently considered symmetric with respect to the ground, [168], some of the commercial RF transformers used in measurements of the IBC system transmission characteristics [15,16,41,155,158,162,163,166,167] did not have symmetrical capacitances between their ‘balanced’ terminals and ground, which highly influenced measurement results [42,153,165]. Most gain measurements in the literature used frequency bandwidth up to 100 MHz, and showed large variance in results, specifically above 50 MHz. However, in [169], the authors claimed that measured gain variance at high frequency range was due to the cables used in measurements and not due to variations in a channel, and they were the first to characterize IBC channel up to 200 MHz using a battery-powered transmitter and a spectrum analyzer as a receiver. One more group investigated wearable capacitive IBC channel in 420–510 MHz frequency range [170] and concluded that such a system was feasible and had low sensitivity to changes of environment, since signals propagate dominantly via surface waves and the return path has a negligible effect on propagation.

Therefore, for performing accurate measurements of IBC channel transmission characteristics, testing apparatuses should be of the same physical size and have the same grounding configurations as devices that will eventually be employed in IBC applications, with the corresponding matching networks between devices and the human body. In other words, measurements of any IBC channel transmission characteristics should be performed using small and independent battery-powered devices, thus bypassing the need for galvanic decoupling and providing a more realistic IBC channel. Removing balun transformers from the measurement setup and using battery-powered devices with very short wires would also inherently allow a wider signal frequency range than in previous measurements. In the case higher frequencies could be exploited for IBC, possibilities for new high data rate applications would open.

#### 3.2.2. IBC Channel Modeling and Device Design

Recent IBC models combined measurements and modeling of all influential parameters in IBC channels, incorporating them in electric circuits and finite-difference time-domain/finite element method (FDTD/FEM) electromagnetic models [150,159,160,171,172,173], in order to better understand wearable or implantable IBC channel and provide guidelines for the design of IBC interfaces and devices.

For on-body devices, in *on-body* to *on-body* (OB2OB) and *in-body* to *on-body* (IB2OB) channels signal and ground electrodes of an IBC devices are usually connected to the body in several ways: (1) both electrodes on the skin (configuration A [41,42], differential [174]), (2) one electrode on the skin, another above it (configuration B [41,42], single-ended [174]), and (3) no direct contact with the skin ([157], non-contact configuration B [41,42]). These impedances have a resistive and capacitive component, which should be measured or estimated for the matching network design. Other impedances between parts of an IBC channel are mostly capacitive, and include [39,159,160]: cross-capacitances between transmitter (TX) and receiver (RX) electrodes, capacitance between electrodes and environment, capacitances between body and environment (40 pF–150 pF [159,160,175,176], 110 pF [177], up to 3.9 pF [178]). All these impedances vary with chosen electrode configuration, environment, and body position, and their influence was proven in measurements of IBC channel gain [15,41,157,173,175,179,180,181,182,183]. For in-body devices (in *in-body* to *in-body* (IB2IB) and *in-body* to *on-body* (IB2OB) channels), parameters of circuit models are difficult to measure but can be extracted from simulation results. Capacitive IBC for implanted devices was simulated using transfer function models which include capacitance between the body and environment [146,147] and FEM models for comparison of capacitive and galvanic implanted electrodes [150].

In recent IBC devices and interfaces, most of the aforementioned parameters were taken into account. Wearable IBC transceiver described in [184] used an auto-loss compensation technique to compensate for the changes in the return signal path due to the body movement, together with the capacitive instead of resistive interface in the receiver. A 4 Mbps data rate with 41 pJ/bit energy efficiency over a 1.5 m distance was achieved. An IBC transceiver was also incorporated in an electroencephalography (EEG) electrode for concurrent EEG signal recording and IBC signal transmission [185]. Furthermore, implantable IBC transceivers were designed: a battery-less 31-µW HBC receiver with RF energy harvester operating at 40 MHz frequency [186] and capacitive IBC transceiver for wireless neural interface applications [187]. The transceiver in [187] achieved a maximum data rate of 16.7 Mb/s with the energy consumptions of 3.7 pJ per received bit and 34 pJ per transmitted bit in the frequency range from DC to 20 MHz.

#### 3.2.3. Capacitive IBC for Communication with In-Body Devices

It was shown recently that a stable capacitive return path could be accomplished not only by exposing the capacitive ground electrode directly to the air (e.g., environment), but also in implantable devices in case the ground electrode was isolated from the human tissue [146,147,148,149,150,171]. Therefore, the capacitive IBC method emerges as an alternative to communication in the Medical Implant Communication Service (MICS) band of IEEE 802.15.6 NB PHY layer [40], UWB communication [188,189] or galvanic coupling intrabody communication [190,191], for implementation of communication with implanted devices, such as endoscopic capsules, pacemakers or cochlear implants [44].

The use of capacitive IBC method for communication between implants was proposed in [146,147]. The authors derived a transfer function of the implant IBC based on capacitive coupling and investigated its characteristics in measurements and simulations. Measurements of transmission characteristics were performed using a battery-powered signal generator, oscilloscope, and a pair of implantable electrodes immersed in a physiological saline solution in a 100 kHz–40 MHz frequency range. Capacitive coupling implantable electrodes consisted of a signal electrode in a direct contact with the physiological solution and a ground electrode covered with a 0.5 mm-thick insulating shell (*σ* = 10^−14^ S/m, *ε_r_* = 3), while the galvanic coupling implantable electrodes were two copper electrodes in a direct contact with the solution. Using the same equipment, higher gain was measured in case of capacitive coupling implantable electrodes and the distance between transmitter and receiver electrodes had almost no influence on the measured gain. Recently, authors from the same group developed a 3-D finite element method model of the human arm incorporating electrode polarization impedance (EPI) and compared results obtained using fully implanted capacitive and galvanic electrodes, [150]. Empirical verification measurements were performed on a porcine tissue using a battery-powered signal generator, a spectrum analyzer and handmade 1 cm-diameter electrodes, in a 100 kHz–100 MHz frequency range. The insulator on the capacitive electrodes was made by using a hot glue polymer. The gain measured using fully implanted capacitive electrodes was 20 dB higher than for a case of using galvanic electrodes at 10 cm transmission distance. The fact that capacitive coupling IBC measured gain minimally depended on a distance between signal and ground electrodes of the same device, while for galvanic coupling-based devices gain increased with distance, suggested that implantable IBC devices based on capacitive coupling could have smaller dimensions than implantable IBC devices based on galvanic coupling.

A group from Korea Advanced Institute of Science and Technology developed a capsule endoscope system with four VGA-resolution cameras [148,149]. Communication between the capsule and on-body receiver was formed by a capacitive coupling via two gold-coated signal electrodes on the transmitter. To increase overall GND coupling (return signal path), the authors proposed increasing the size of the metallic GND plane integrated in the receiver. Intrabody communication performance was tested between approximately 10 MHz and 400 MHz using their proprietary endoscopic capsule and a battery-powered receiver power detector. The human torso was simulated using a diacetin-based tissue-mimicking phantom in a water tank [192], while the skin-to-electrode interface was emulated with abdominal pigskin placed between the phantom and the receiver. The capsule with IBC transmitter was located inside of pig small intestine and immersed in the phantom solution. The measured channel characteristic gradually increased up to 100 MHz and maintained a flat band up to 200 MHz. The worst case scenario of capsule signal electrodes not touching the intestine tissue was also tested. A few centimeters of separation degraded the measured gain by 10 dB, but the reliable communication was nevertheless achieved.

Since experiments with implanted capacitive IBC devices on living beings would be highly invasive, measurements of implantable capacitive IBC channels are usually made on human body phantoms rather than on humans. The tissue phantom in which the in-body transmitter is placed needs to be liquid, so the distance between transmitter and receiver electrodes can be adjusted during the measurements; while outer tissues in multilayer phantoms can be semi-solid or animal skin as in [148,149]. Receiver electrodes are placed inside the phantom for IB2IB channel measurements, and on the outer layer of a phantom for IB2OB channel measurements.

Human tissue phantoms are made combining simple chemical substances with water for adjusting conductivity and relative permittivity of the solution. However, it is rather difficult to produce solutions that emulate the electrical properties of human tissues in a wide range of frequencies [192,193], so a single phantom can be used at specified frequency or in a narrow frequency band. Human body phantoms described in the literature were mostly intended for microwave frequencies [193] and there were only few papers dealing with recipes for tissue-equivalent phantoms below 200 MHz, especially for tissues other than muscle [192,194,195,196,197,198,199]. Ingredients for muscle phantom preparation were usually water, sodium chloride, and aluminum powder (13.56–100 MHz; to increase permittivity of aqueous solution) or polyethylene powder (200–2450 MHz; to reduce permittivity) [194]. Liquid phantoms are exposed to evaporation so their electric parameters might change over time and need to be checked regularly. For increasing viscosity and achieving a semisolid phantom, a gelling agent like TX-150 was used. Measurements of capacitive implantable IBC channel characteristics were made using battery-powered equipment on a physiological saline solution [146,147] and liquid diacetin-based phantom [148,149], as well as on the porcine tissue, since it has similar electrical properties as the human tissue [150]. Preliminary results of the first in vivo measurements of capacitive intrabody communication with implant-like devices on humans were presented in [200]. The authors mimicked an IB2OB channel by placing the transmitter under the armpit and taking different body positions, while covering transmitter electrodes with tissue. The results agreed qualitatively to results of the OB2OB channel measurements obtained using the same battery-powered equipment and baluns for decoupling as in [41,42]. Other promising on-body locations for emulating IB2OB channel, where transmitter electrodes can be covered with tissue are e.g., inside mouth, under the armpit, and behind the knee. However, for obtaining reliable results these measurements should be repeated using small battery-powered devices and tested on a large group of individuals, in order to analyze the influence of anthropometric properties of a subject. It is expected that in capacitive IB2OB and IB2IB channels an intersubject variability (anthropometrical and bioelectric properties of a subject) will have higher impact on measurement results than in the case of a capacitive OB2OB channel (for which it is almost negligible [13]), as is the case with galvanic IBC channels [15,180,201].

## 4. Comparison, Discussion and Open Challenges: UWB and IBC Technologies

This paper provides an in-depth overview of two emerging technologies for WBAN applications, ultra-wideband (UWB) and intrabody communication (IBC), respectively. Both technologies have their own distinctive advantages over the traditional wireless communication approaches, with a potential to provide new range of solutions that could exploit particular benefits of each technology. This paper aimed to provide an extensive overview of capabilities and comparison of relevant standards for UWB and IBC technologies in modern WBAN solutions. There are other overviews of similar topics in the literature [202,203,204], where the state of the art in WBAN is presented. However, unlike them, this paper focused on the overview of UWB and IBC technologies applications in previous few years, with emphasis on the overview of UWB technology in healthcare and human motion tracking along with the useful theoretical background overview of the UWB communication and signal propagation modeling, as well as an in-depth overview of the capacitive IBC for implantable channels. Therefore, this paper could be useful as a reference paper for understanding of the UWB and IBC theoretical background as well as an overview of the studies in the aforementioned topics in the past few years.

Both technologies address issues of the efficient and standardized interconnection of on-body, off-body, and in-body sensors and devices in WBANs. Some of advantages of UWB-based systems are immunity to fading, reduced power spectral density, and interference with other communication systems, simple hardware design, centimeter-level local positioning, extremely fine time resolution, high-data rates, capability of simultaneous sensing and data communication, low power, and low-cost. UWB systems are especially promising technology in the area of healthcare, providing new possibilities of addressing problems, such as vital signs monitoring, breast tumor detection, wireless capsule endoscopy, precise static and dynamic human motion tracking, and radar-based remote sensing and imaging. The most important IBC advantages are very low power consumption due to the low operating frequency, immunity to interferences from external RF devices, confinement of communication in the close proximity of the human body, thus providing higher security by reducing the risk of eavesdropping, and possibility of system implementation by using simple electrodes instead of RF antennas. Both technologies are safe for use in WBANs in an environment where devices are very closely coupled with the human body.

Such favorable characteristics of UWB and IBC technologies lead to the extensive research about the possibilities of their use in a wide range of application areas. However, these technologies have not been yet adapted in everyday use and commercial devices because they are both still under intensive research and, therefore, relevant standards that regulate their use have appeared only recently. This paper provides a detailed overview of the state of the art and the most important guidelines of present standards IEEE 802.15.4 (for UWB) and IEEE 802.15.6 (for UWB and IBC). These standards are important as they provide a ground for real-world adoption of these new technologies that have a potential to introduce advanced novel solutions in various application fields, such as healthcare, sports, and entertainment. Complying with standards might also expedite medical sensor development and shorten the duration of the medical agencies’ approval.

However, both technologies have their respective limitations, mostly due to variations in human body as the signal propagation channel, influence of random body movements, and challenges of UWB antenna and IBC electrodes design due to uncertainty of parameters of signal propagation model. These issues are the most important topics of the current research efforts that aim to overcome the obstacles towards wider adoption of these technologies in real-world applications.

Due to all aforementioned advantages and good characteristics, the combination of UWB and IBC could be an excellent candidate technology for future IB2OB communications in next-generation WBANs. Low power consumption of both UWB and IBC would provide even more power efficient solutions that could make use not only of the energy stored in batteries, but also of the energy harvesting approaches. Capacitive IBC is a particularly interesting solution to replace wires and RF communication for in-body devices considering low-frequency operation and, consequently, better path-loss characteristics than UWB. IBC’s further advantage is that it can be realized only by using small electrodes, without a need for antennas, which are typically prone to the unpredictable influence of the nearby human body. Lots of efforts in IBC research are directed to the capacitive IBC for implantable channels, what is currently a hot research topic. On the other hand, UWB is particularly well-suited for some specific applications, such as OB2OB for contactless vital signs monitoring (even through obstacles in NLOS conditions), OB2OFF for precise human tracking motion (as a part of real-time localization system, RTLS), and wireless capsule endoscopy (use case for IB2OB scenario).

## Figures and Tables

**Table 1:** Summary of UWB vital signs monitoring application reported in the literature.

1st Author Year	Ref	Parameter	Method	UWB System (Frequency)	Application Scenario	Accuracy (Limitation)
Nguyen, 2014	[76]	HR, RR	SHAPA algorithm	IR-UWB radar Central freq. 4.1 GHz	Eight subjects, lying on the top of a mattress	Practical for a real-time system
Brovoll, 2014	[78]	Human Heart Motion	Time-lapse imaging	Switched Array UWB r. 0.75–2.27 GHz	Antenna was aligned with the body	The subject was holding his breath
Hu, 2016	[79]	HR, RR	EEMD;CWT	IR-UWB radar Central freq. 6.8 GHz	Sitting in a chair and breathing regularly	SNR of RR and HR improved by 7.59 dB and 4.82 dB
Ren, 2016	[80]	HR	Phase-Based	UWB Impulse Doppler r. 1.5–4.5 GHz	Subject sat still in front of the radar system	CSD and AD heart rate deviation is 2.6% and operating rate is 0.8 m
Yin, 2016	[47]	HR	Cascade CNN	NVA-R661 IR-UWB radar module	HR analysis by combination of the ECG and radar	Results accuracy of 88.89% in the slight motion state
Shy, 2018	[82]	HR, RR	FVPIEF based 2-Layer EEMD	UWB radar Central freq. 4.3 GHz	Simultaneously analysis of RR and HR	Relatively accurately
Shen,2018	[22]	HR, RR Subject location	Autocorr. VMD; FFT	PulsOn410 UWB radar Center freq. 4.3 GHz	Vital signs monitoring	Potential implemention in integrated circuits and embedded systems
Yim, 2019	[83]	HR Subject position	CFAR algorithm	XK300-MVI radar 7.29–8.748 GHz	Clinical application	Quantified index to clinically record
Kim, 2019	[84]	RR	1D CNN model	UWB radar 3–4 GHz	Eupnea, bradypnea, tachypnea, apnea, and motion classification	Average recognition rate of respiration patterns 93.9%

**Table 2:** Comparison of ultra-wideband communication systems in WBAN reported in literature.

1st Author Year	Ref	Application	System	Frequency Band	Data Rate	Features
Garcia-Pardo, 2016	[141]	UWB path loss models and channel measurements for IB2OB and IB2OFF communication scenarios	Two UWB omnidirectional patch antennas	3.1–8.5 GHz	20001 frequency points	Practical estimation of UWB transmissions path loss from wireless devices implanted in the abdominal cavity to an external unit Implantable application
Kjelgård, 2017	[12]	Heart Wall Velocity Sensing	Body coupled antennas, RF-amplifiers, core radar processor (Novelda X2)	Center freq. 4 GHz	>35 GS/s	Good correlation with tissue doppler ultrasound and microwave radar
Leelatien, 2018	[3]	Wireless monitoring of transplanted organs (liver)	A low-profile tapered UWB antenna with vertical polarization	4.5–6.5 GHz	10 Mb/s	Attenuation variation 30 dB (with respect to 40 mm largest organ movement distance) due to respiration-induced organ movement
Schires, 2018	[24]	Through the back vital signs monitoring	UWB Novelda Xethru X2 chip with body coupled antennas	3.8–9 GHz	65 frames per second	High accuracy of back monitoring of vital sign using a pulsed radar mounted into a car seat
Zhang, 2018	[142]	Vital signs radar sensing and short-range communication and	CMOS IR – UWB radar and communication interface	BW = 5.6	10 Mb/s	Power consumption 6.4 mW and sensitivity −64 dBm at 10 Mb/s
Perez-Simbor, 2019	[88]	Wireless capsule endoscopy	Quasi-omnidirectional antenna	3.1–8.5 GHz (phantom) 3.1–6 GHz (in-vivo)	Resolution point:3201	Study of the path loss using simulations, phantoms, and in-vivo measurements
Han, 2019	[107]	In-body to on-body links	HBC-UWB signals Simulation	10–50 MHz		Combination of HBC and UWB band signal, better high data rate
Lauteslager, 2019	[34]	Measurement of Cardiovascular Dynamics	UWB Xethru X2 single-chip radar in combination with body coupled antennas	BW = 2.5 GHz	64 frames per second	High accuracy
Särestöniemi, 2019	[7]	Human abdomen area UWB on-body radio channel characteristics	UWB on body antenna (measurements conducted in an anechoic chamber)	3.75–4.25 GHz		If the antenna separation distance is large on-body channel characteristics vary significantly depending on the body size and shape
Fang, 2019	[9]	Channel modeling inside a UWB liquid phantom for wireless capsule endoscopy	Planar elliptical ring implanted IB antenna Semicircle monopole OB antenna	3.1–5.1 GHz	Frequency point 1601	Proposed models confirm the agreement with the radiation performance of the designed in-body antenna
Song, 2019	[28]	Breast tumors detection	CMOS-IC portable IR-UWB-radar	0.5–20 GHz	100 G Sample/s	Results: IR-UWB radar-based detector has a potential for early-stage breast cancers detection
Kumpuniemi, 2019	[108]	Dynamic off-body on UWB frequencies radio channels measurements	Two planar prototype (dipole and double loop) antennas Six on-body antenna locations, one off-body site	2–8 GHz	1601 points in the band	Mean path losses varied between 47.6–69.4 dB (average distance of 2 m)
Särestöniemi, 2020	[30]	UWB radio channel characteristics study between a capsule endoscope and a directive on-body antenna in different parts of the small intestine	In-body omnidirectional dipole antenna (capsule model) from [143] Two on-body directive low-band UWB antennas from [106,144]	In-body antenna 4 GHz On-body antennas 3.75–4.25 GHz		Radio channel characteristics varied significantly depending on the capsule model and on-body antennas location Investigation of the power flow: influence of the cavity sizes

## Abbreviations

BCC	Body channel communication
BPM	Burst position modulation
BPSK	Binary phase shift keying
BS	Base station
BT	Bluetooth
CIR	Channel impulse response
CSS	Chirp spread spectrum
DS-CDMA	Direct-Sequence Code Division Multiple Access
DS-UWB	Direct-Sequence UWB
ECC	European Communication Committee
ECG	Electrocardiograph
EIRP	Equivalent isotropically radiated power
EPI	Electrode polarization impedance
EMC	Electromagnetic compatibility
EMG	Electromyography
FCC	Federal Communications Commission
FDTD	Finite difference time-domain
FEM	Finite element method
GI	Gastrointestinal
GPR	Ground penetrating radars
HBC	Human body communication
HMT	Human motion tracking
HR	Heart rate
IB2IB	In-body to in-body
IB2OB	In-body to on-body
IBC	Intrabody communication
IEEE	Institute of Electrical and Electronics Engineers
IMU	Inertial measurement unit
IR-UWB	Impulse radio UWB
LOS	Line of sight
MAC	Medium Access Control
MBOFDM	Multiband orthogonal frequency division multiplexing
MB-UWB	multiband UWB
MICS	Medical Implant Communication Service
MPC	Multipath components
NLOS	Non-line of sight
OB2OB	On-body to on-body
PDP	Power delay profile
PHY	Physical layer
PL	Path loss
PRF	Pulse repetition frequency
PSD	Power spectral density
QoS	Quality-of-Service
RBM	Random body movement
RF	Radio frequency
RR	Respiration rate
RX	Receiver
SV	Saleh-Valenzuela
TH	Time-hopping
TOA	Time of arrival
TX	Transmitter
UWB	Ultra-wideband
WBAN	Wireless body area network
WBS	Wireless body sensors
WLAN	Wireless LAN
WPAN	Wireless personal area networks
WSN	Wireless sensor network
